# Heat vs. hue: A molecular tug-of-war in pear fruit coloration

**DOI:** 10.1093/plcell/koaf100

**Published:** 2025-05-02

**Authors:** Nitin Uttam Kamble

**Affiliations:** Assistant Features Editor, The Plant Cell, American Society of Plant Biologists; Indian Institute of Science Education and Research, Thiruvananthapuram 695551, India

Fruits are daily alchemy; every morning, we peel back nature's palette and consume its colors: yellow bananas, orange slices, green kiwis, fiery red apples, golden mangoes, and deep violet grapes. In every fruit lies a language spoken not in words but in color. In a life often dulled by repetition and routine, fruits quietly and beautifully remind us that nature still makes art.

This colorful artwork is the result of an accumulation of compounds, such as anthocyanins, which provide red pigmentation in fruits. High temperatures adversely affect fruit development and quality, including fruit color, by impacting anthocyanin biosynthesis in fruits. Red pears are priced for their attractive pigmentation in many parts of the world. Although genes and transcriptional cascades involved in fruit pigmentation are well understood, the inhibition of anthocyanin accumulation under high temperatures and the underlying molecular mechanism in pear remains unexplored (Bai et al. 2019; Su et al. 2022; Ni et al. 2023).

In new work, **Lu Wang and colleagues (Wang et al. 2025)** provide a thorough analysis of high-temperature–induced inhibition of anthocyanin accumulation in pear fruit. After confirming the negative effects of high temperature on anthocyanin levels and related biosynthetic genes in both whole fruit and callus tissues, Wang et al. conducted deep transcriptome sequencing (RNA-seq) coupled with weighted gene coexpression network analysis to uncover the molecular mechanisms behind this inhibition.

The authors identified *Pp*HY5L, a transcription factor containing a bZIP HY5-like domain, as a positive regulator of anthocyanin biosynthesis that is repressed under high-temperature conditions along with other transcription factors of anthocyanin biosynthesis such as *Pp*MYBL, *Pp*TT2, and *Pp*WRKY75. Although HY5 is known to play a role in anthocyanin regulation, high temperature had minimal effect on *PpHY5* expression, suggesting that other mechanisms may interfere with HY5 function (Bai et al. 2019).

Next, Wang et al. screened the *PpHY5L* promoter and identified a heat shock element in *PpHY5L* promoter sequence (Fig.). Heat Shock Factors (HSFs), which bind to heat shock elements, are well-known regulators of heat responses in plants (Mittler et al. 2012;Wang et al. 2020). Using the PlantTFDB (https://planttfdb.gao-lab.org/) and RNA-seq data, the authors identified *Pp*HsfB2a as a candidate transcription factor whose expression was induced by high temperatures. The authors also observed that *Pp*HsfB2a could regulate its own expression under prolonged heat exposure. Using yeast 1-hybrid assays, electrophoretic mobility shift assays, chromatin immunoprecipitation–quantitative PCR, and dual-luciferase assays, Wang et al. confirmed their hypothesis that *Pp*HsfB2a physically binds to *PpHY5L* promoter and represses *PpHY5L* expression (Fig.). Heat-induced *Pp*HsfB2a expression may thus be at the basis of heat-regulated and *PpHY5L-*mediated fruit coloration.

Functional characterization of *PpHsfB2a* through RNAi silencing and overexpression in a stable pear callus transformation system confirmed that *Pp*HsfB2a acts as a negative regulator of anthocyanin biosynthesis. For instance, *PpHsfB2a RNAi* calli showed intense red coloration and accumulated more anthocyanins, while calli overexpressing *PpHsfB2a* had reduced anthocyanin levels correlating with reduced *PpHY5L* transcript levels.

Wang et al. further examined the stability of the *Pp*HsfB2a protein at different temperatures. Using a cell-free degradation assay, the authors showed that *Pp*HsfB2a levels are maintained at higher temperatures. Immunoprecipitation followed by mass spectrometry identified a RING-type E3 ubiquitin ligase, *Pp*ATL52, similar to Arabidopsis ATL52 (TÓXICOS EN LEVADURA 52) as a regulator of *Pp*HsfB2a stability. Using a range of in vivo and in vitro methods, including functional characterization in pear, the authors demonstrated that *Pp*ATL52 interacts with *Pp*HsfB2a, mediating its ubiquitination and degradation, thereby modulating anthocyanin accumulation (Fig.).

In summary, this study elucidates previously unknown mechanisms of high-temperature–induced inhibition of anthocyanin accumulation in pear, revealing a *Pp*ATL52–*Pp*HsfB2a–*Pp*HY5L regulatory module. Future investigations are needed to determine the relationship between heat stress, reactive oxygen species, and anthocyanin biosynthesis, as well as how light and temperature jointly influence *Pp*ATL52 regulation. Further studies are also required to explore the roles in pear pigmentation of additional transcription factors identified in this work, such as *Pp*MYBL, *Pp*TT2, and *Pp*WRKY75. Understanding these regulatory mechanisms carries implications for developing fruit varieties with improved color.

**Figure.**
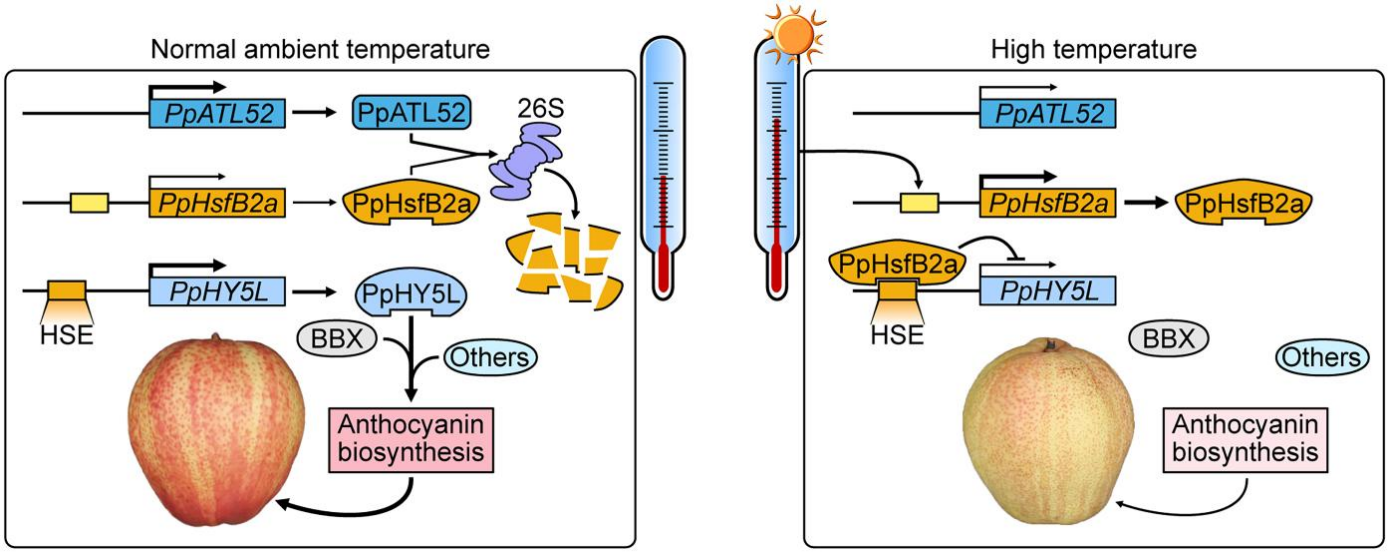
High temperature inhibits anthocyanin biosynthesis in pear. Under ambient temperature (20 °C), *Pp*ATL52 degrades *Pp*HsfB2a via the 26S proteasome pathway, reducing the inhibitory effect on *Pp*HY5L expression and inducing the expression of anthocyanin synthesis genes. However, at higher temperature (35 °C), lower expression of *Pp*ATL52 and reduced degradation of *Pp*HsfB2a suppress *Pp*HY5L expression, inhibiting anthocyanin biosynthesis. Reprinted from Wang et al. (2025), Figure 8.

## Recent related articles in *The Plant Cell*:

- Sun et al. (2025) reports function of *Vv*FHY3 during high temperatures linking auxin and endoplasmic reticulum stress to regulate grape anthocyanin biosynthesis.
- Wei et al. (2023) reports high-temperature–induced repression of chlorophyll catabolism by proteasomal degradation of *Ma*MYB60 mediated by the E3 ligase *Ma*BAH1 in banana.
- Zhang et al. (2024) reports on ROS-dependent anthocyanin biosynthesis in pear by phosphorylated transcription factor *Pu*HB40 when exposed to high light.
- Ni et al. (2023) reports on inhibition of anthocyanin biosynthesis via histone deacetylation-mediated repression of *Pp*RAP2.4 and *Pp*MYB114 by ethylene-responsive transcription factor in pear.
